# Crystal structure of *N*-{[3-bromo-1-(phenyl­sulfon­yl)-1*H*-indol-2-yl]meth­yl}benzene­sulfonamide

**DOI:** 10.1107/S2056989015016874

**Published:** 2015-09-17

**Authors:** M. Umadevi, P. Raju, R. Yamuna, A. K. Mohanakrishnan, G. Chakkaravarthi

**Affiliations:** a Research and Development Centre, Bharathiar University, Coimbatore 641 046, India; b Department of Chemistry, Pallavan College of Engineering, Kanchipuram 631 502, India; c Department of Organic Chemistry, University of Madras, Guindy Campus, Chennai 600 025, India; d Department of Sciences, Chemistry and Materials Research Lab, Amrita Vishwa Vidyapeetham University, Ettimadai, Coimbatore 641 112, India; e Department of Physics, CPCL Polytechnic College, Chennai 600 068, India

**Keywords:** crystal structure, benzene­sulfonamide, phenyl­sulfon­yl, bio­logical activity, derivatives, hydrogen bonding, C—H⋯π inter­actions

## Abstract

In the title compound, C_21_H_17_BrN_2_O_4_S_2_, the indole ring system subtends dihedral angles of 85.96 (13) and 9.62 (16)° with the planes of the N- and C-bonded benzene rings, respectively. The dihedral angles between the benzene rings is 88.05 (17)°. The mol­ecular conformation is stabilized by intra­molecular N—H⋯O and C—H⋯O hydrogen bonds and an aromatic π–π stacking [centroid-to-centroid distance = 3.503 (2) Å] inter­action. In the crystal, short Br⋯O [2.9888 (18) Å] contacts link the mol­ecules into [010] chains. The chains are cross-linked by weak C—H⋯π inter­actions, forming a three-dimensional network.

## Related literature   

For the biological activity of indole derivatives, see: Andreani *et al.* (2001 ▸); Andreev *et al.* (2015 ▸); Kolocouris *et al.* (1994 ▸). For related structures, see: Chakkaravarthi *et al.* (2007 ▸, 2008 ▸). 
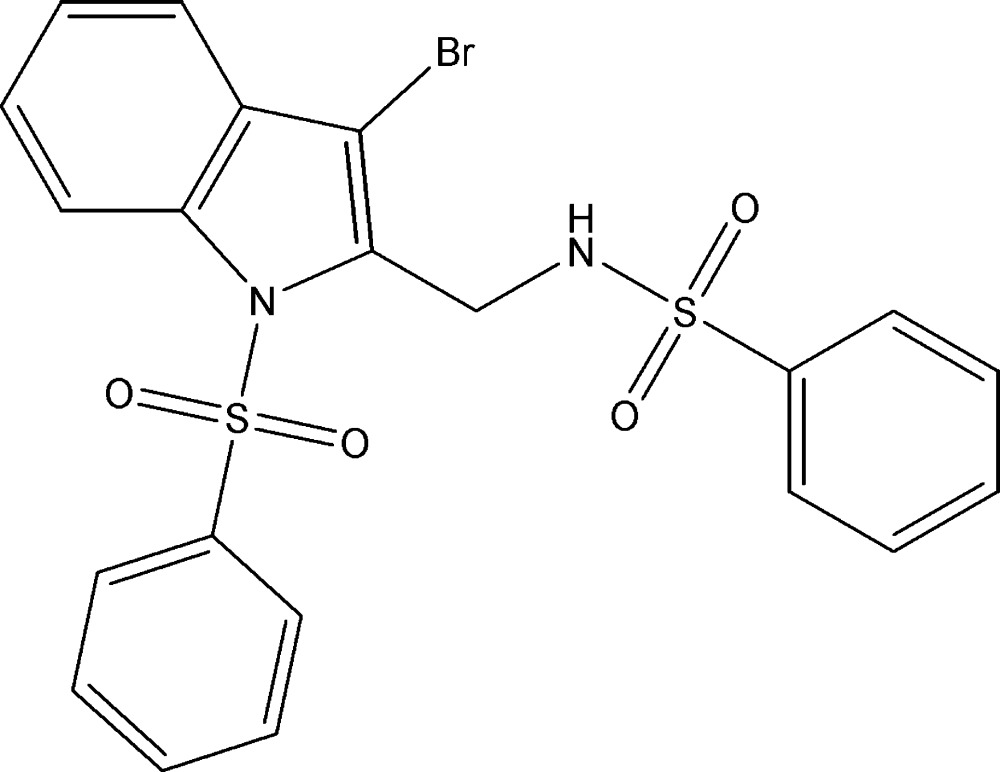


## Experimental   

### Crystal data  

C_21_H_17_BrN_2_O_4_S_2_
*M* *_r_* = 505.40
Monoclinic, 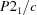

*a* = 7.5129 (6) Å
*b* = 17.4245 (14) Å
*c* = 16.0988 (14) Å
β = 98.087 (3)°
*V* = 2086.5 (3) Å^3^
*Z* = 4
Mo *K*α radiation
μ = 2.20 mm^−1^
*T* = 295 K
0.28 × 0.24 × 0.22 mm

### Data collection  

Bruker Kappa APEXII CCD diffractometer
Absorption correction: multi-scan (*SADABS*; Sheldrick, 1996 ▸) *T*_min_ = 0.578, *T*_max_ = 0.643
23680 measured reflections
3808 independent reflections
2942 reflections with *I* > 2σ(*I*)
*R*_int_ = 0.042

### Refinement  

*R*[*F* ^2^ > 2σ(*F* ^2^)] = 0.036
*wR*(*F* ^2^) = 0.100
*S* = 1.02
3808 reflections
275 parameters
1 restraint
H atoms treated by a mixture of independent and constrained refinement
Δρ_max_ = 0.60 e Å^−3^
Δρ_min_ = −0.40 e Å^−3^

### 

Data collection: *APEX2* (Bruker, 2004 ▸); cell refinement: *SAINT* (Bruker, 2004 ▸); data reduction: *SAINT*; program(s) used to solve structure: *SHELXS97* (Sheldrick, 2008 ▸); program(s) used to refine structure: *SHELXL97* (Sheldrick, 2008 ▸); molecular graphics: *PLATON* (Spek, 2009 ▸); software used to prepare material for publication: *SHELXL97* and *PLATON*.

## Supplementary Material

Crystal structure: contains datablock(s) global, I. DOI: 10.1107/S2056989015016874/hb7501sup1.cif

Structure factors: contains datablock(s) I. DOI: 10.1107/S2056989015016874/hb7501Isup2.hkl

Supporting information file. DOI: 10.1107/S2056989015016874/hb7501Isup3.cml

**Fig. 1.**
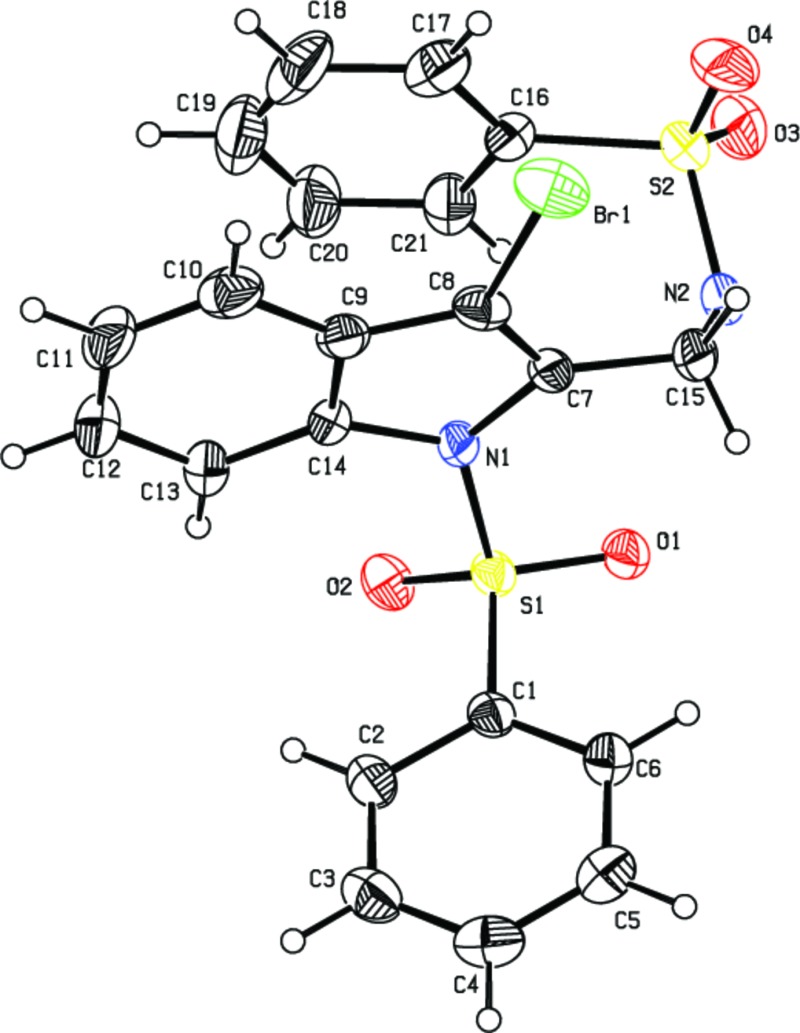
The molecular structure of (I), with 30% probability displacement ellipsoids for non-H atoms.

. DOI: 10.1107/S2056989015016874/hb7501fig1.tif
The mol­ecular structure of (I), with 30% probability displacement ellipsoids for non-H atoms.

CCDC reference: 1423139

Additional supporting information:  crystallographic information; 3D view; checkCIF report

## Figures and Tables

**Table 1:** Hydrogen-bond geometry (, ) *Cg*2 is the centroid of the C1C6 ring.

*D*H*A*	*D*H	H*A*	*D* *A*	*D*H*A*
N2H2*A*O1	0.87(1)	2.17(3)	2.795(3)	128(3)
C13H13O2	0.93	2.38	2.954(4)	120
C21H21*Cg*2^i^	0.93	2.81	3.667(3)	155

Symmetry code: (i) 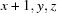.

## supplementary crystallographic information

### S1. Comment

Indole derivatives are known to exhibit activities such as antitumour (Andreani *et al.*, 2001); anti-hepatitis C virus (Andreev *et al.*, 2015) and antiviral (Kolocouris *et al.*, 1994). We herein report the crystal structure of (I). The *ORTEP* diagram of the title compound (I) is shown in Fig. 1. The geometric parameters of (I) are comparable with the reported similar structures (Chakkaravarthi *et al.* 2007, 2008). The phenyl rings (C1—C6) and (C16—C21) form the dihedral angles of 85.96 (13)° and 9.62 (16)°, respectively with the indole ring system. The phenyl rings (C1—C6) and (C16—C21) are orthogonal to each other, with the dihedral angle of 88.05 (17)°.

The molecular structure (Fig. 1) is stabilized by weak intramolecular N—H···O, C—H···O and C—H···Br hydrogen bonds (Table 1) and π–π [centroid-to-centroid distance = 3.503 (2) Å] interaction. In the crystal structure, the intermolecular weak Br···O [Br1···O2^i^ and O2··· Br1^ii^ contacts at 2.99 Å; (i) (1 - *x*,-1/2 + *y*,1/2 - *z*); (ii) 1 - *x*,1/2 + *y*,1/2 - *z*)] contacts form the molecules into infinite one-dimensional chain along [0 1 0] and these chains are further influenced by weak C—H···π interactions (Table 1) to form a three dimensional network.

### S2. Experimental

To a solution of 3-bromo-2-(bromomethyl)-1-(phenylsulfonyl)-1*H*-indole (0.5 g, 1.16 mmol) in acetonitrile (10 ml) was added K_2_CO_3_ (0.32 g, 2.33 mmol) and benzenesulphonamide (0.23 g, 1.51 mmol). The reaction mixture was then stirred at reflux for 12 h. After completion of the reaction (monitored by TLC), it was cooled to room temperature and reaction mass was poured over crushed ice (30 g). The solid obtained was filtered and dried. The crude product was recrystallized from methanol to afford the title compound as colourless blocks.

### S3. Refinement

H atoms were positioned geometrically and refined using riding model, with C—H = 0.93 Å and *U*_iso_(H) = 1.2Ueq(C) for C—H and C—H = 0.97 Å and *U*_iso_(H) = 1.2Ueq(C) for CH_2_. H atom for N atom is fixed from Fourier map and refined freely with distance restraint of 0.88 (1) Å. The reflection (0 1 1) is omitted during refinement which is owing poor agreement.

### Crystal data

**Table:**

C_21_H_17_BrN_2_O_4_S_2_	*F*(000) = 1024
*M**_r_* = 505.40	*D*_x_ = 1.609 Mg m^−^^3^
Monoclinic, *P*2_1_/*c*	Mo *K*α radiation, λ = 0.71073 Å
Hall symbol: -P 2ybc	Cell parameters from 7461 reflections
*a* = 7.5129 (6) Å	θ = 2.3–24.4°
*b* = 17.4245 (14) Å	µ = 2.20 mm^−^^1^
*c* = 16.0988 (14) Å	*T* = 295 K
β = 98.087 (3)°	Block, colourless
*V* = 2086.5 (3) Å^3^	0.28 × 0.24 × 0.22 mm
*Z* = 4	

### Data collection

**Table:**

Bruker Kappa APEXII CCD diffractometer	3808 independent reflections
Radiation source: fine-focus sealed tube	2942 reflections with *I* > 2σ(*I*)
Graphite monochromator	*R*_int_ = 0.042
ω and φ scan	θ_max_ = 25.4°, θ_min_ = 2.3°
Absorption correction: multi-scan (*SADABS*; Sheldrick, 1996)	*h* = −9→8
*T*_min_ = 0.578, *T*_max_ = 0.643	*k* = −20→20
23680 measured reflections	*l* = −19→19

### Refinement

**Table:**

Refinement on *F*^2^	Primary atom site location: structure-invariant direct methods
Least-squares matrix: full	Secondary atom site location: difference Fourier map
*R*[*F*^2^ > 2σ(*F*^2^)] = 0.036	Hydrogen site location: inferred from neighbouring sites
*wR*(*F*^2^) = 0.100	H atoms treated by a mixture of independent and constrained refinement
*S* = 1.01	*w* = 1/[σ^2^(*F*_o_^2^) + (0.055*P*)^2^ + 0.8751*P*] where *P* = (*F*_o_^2^ + 2*F*_c_^2^)/3
3808 reflections	(Δ/σ)_max_ < 0.001
275 parameters	Δρ_max_ = 0.60 e Å^−^^3^
1 restraint	Δρ_min_ = −0.40 e Å^−^^3^

### Special details

**Table:**

Geometry. All e.s.d.'s (except the e.s.d. in the dihedral angle between two l.s. planes) are estimated using the full covariance matrix. The cell e.s.d.'s are taken into account individually in the estimation of e.s.d.'s in distances, angles and torsion angles; correlations between e.s.d.'s in cell parameters are only used when they are defined by crystal symmetry. An approximate (isotropic) treatment of cell e.s.d.'s is used for estimating e.s.d.'s involving l.s. planes.
Refinement. Refinement of *F*^2^ against ALL reflections. The weighted *R*-factor *wR* and goodness of fit *S* are based on *F*^2^, conventional *R*-factors *R* are based on *F*, with *F* set to zero for negative *F*^2^. The threshold expression of *F*^2^ > σ(*F*^2^) is used only for calculating *R*-factors(gt) *etc*. and is not relevant to the choice of reflections for refinement. *R*-factors based on *F*^2^ are statistically about twice as large as those based on *F*, and *R*- factors based on ALL data will be even larger.

### Fractional atomic coordinates and isotropic or equivalent isotropic displacement parameters (Å^2^)

**Table:**

	*x*	*y*	*z*	*U*_iso_*/*U*_eq_	
C1	0.2432 (4)	0.19281 (14)	0.09825 (16)	0.0381 (6)	
C2	0.1728 (4)	0.25496 (16)	0.13721 (19)	0.0481 (7)	
H2	0.2437	0.2827	0.1788	0.058*	
C3	−0.0038 (4)	0.27433 (19)	0.1127 (2)	0.0594 (8)	
H3	−0.0543	0.3151	0.1384	0.071*	
C4	−0.1065 (4)	0.2335 (2)	0.0501 (2)	0.0611 (9)	
H4	−0.2255	0.2476	0.0332	0.073*	
C5	−0.0355 (4)	0.1724 (2)	0.0126 (2)	0.0601 (8)	
H5	−0.1065	0.1450	−0.0293	0.072*	
C6	0.1410 (4)	0.15136 (17)	0.03663 (18)	0.0498 (7)	
H6	0.1900	0.1098	0.0115	0.060*	
C7	0.4610 (4)	0.02955 (14)	0.20280 (17)	0.0396 (6)	
C8	0.4202 (4)	0.00661 (15)	0.27698 (19)	0.0460 (7)	
C9	0.4154 (4)	0.07015 (17)	0.33242 (18)	0.0453 (7)	
C10	0.3906 (5)	0.0770 (2)	0.4160 (2)	0.0625 (9)	
H10	0.3682	0.0340	0.4471	0.075*	
C11	0.4000 (5)	0.1484 (3)	0.4513 (2)	0.0736 (11)	
H11	0.3829	0.1540	0.5071	0.088*	
C12	0.4344 (5)	0.2125 (2)	0.4059 (2)	0.0692 (10)	
H12	0.4376	0.2605	0.4314	0.083*	
C13	0.4638 (4)	0.20715 (18)	0.32402 (18)	0.0544 (8)	
H13	0.4891	0.2503	0.2938	0.065*	
C14	0.4545 (4)	0.13474 (16)	0.28803 (16)	0.0408 (6)	
C15	0.5031 (4)	−0.01861 (16)	0.13068 (18)	0.0494 (7)	
H15A	0.4799	−0.0719	0.1430	0.059*	
H15B	0.4206	−0.0045	0.0812	0.059*	
N2	0.6852 (4)	−0.01280 (14)	0.11069 (15)	0.0499 (6)	
C16	0.8919 (4)	0.02153 (17)	0.26010 (18)	0.0480 (7)	
C17	0.8597 (5)	−0.0015 (2)	0.3376 (2)	0.0676 (9)	
H17	0.8240	−0.0515	0.3468	0.081*	
C18	0.8818 (6)	0.0519 (4)	0.4024 (2)	0.0907 (15)	
H18	0.8611	0.0378	0.4559	0.109*	
C19	0.9337 (6)	0.1249 (3)	0.3874 (3)	0.0932 (15)	
H19	0.9473	0.1604	0.4310	0.112*	
C20	0.9656 (6)	0.1467 (2)	0.3107 (3)	0.0802 (11)	
H20	1.0023	0.1966	0.3017	0.096*	
C21	0.9440 (5)	0.09537 (18)	0.2461 (2)	0.0615 (9)	
H21	0.9645	0.1103	0.1928	0.074*	
N1	0.4842 (3)	0.11047 (11)	0.20700 (13)	0.0390 (5)	
O1	0.5276 (3)	0.12773 (11)	0.05899 (11)	0.0453 (5)	
O2	0.5650 (3)	0.23686 (10)	0.15614 (13)	0.0495 (5)	
O3	1.0073 (3)	−0.03343 (15)	0.12907 (15)	0.0726 (7)	
O4	0.8142 (4)	−0.11538 (13)	0.20432 (18)	0.0801 (8)	
S1	0.47029 (9)	0.16993 (3)	0.12591 (4)	0.03756 (17)	
S2	0.85938 (11)	−0.04228 (4)	0.17424 (5)	0.0547 (2)	
Br1	0.39126 (5)	−0.095232 (19)	0.30887 (3)	0.07287 (16)	
H2A	0.709 (4)	0.0315 (11)	0.0891 (19)	0.063 (10)*	

### Atomic displacement parameters (Å^2^)

**Table:**

	*U*^11^	*U*^22^	*U*^33^	*U*^12^	*U*^13^	*U*^23^
C1	0.0491 (15)	0.0309 (13)	0.0358 (14)	0.0022 (11)	0.0105 (12)	0.0067 (11)
C2	0.0550 (18)	0.0405 (15)	0.0503 (17)	0.0039 (13)	0.0131 (14)	−0.0015 (13)
C3	0.060 (2)	0.0540 (19)	0.068 (2)	0.0136 (15)	0.0194 (17)	0.0043 (16)
C4	0.0473 (18)	0.074 (2)	0.063 (2)	0.0076 (16)	0.0136 (16)	0.0215 (18)
C5	0.057 (2)	0.070 (2)	0.0511 (19)	−0.0028 (16)	−0.0004 (15)	0.0005 (16)
C6	0.0585 (18)	0.0481 (16)	0.0429 (16)	0.0051 (14)	0.0071 (14)	−0.0033 (13)
C7	0.0458 (15)	0.0278 (13)	0.0426 (16)	0.0014 (11)	−0.0027 (12)	−0.0019 (11)
C8	0.0471 (16)	0.0343 (14)	0.0553 (18)	0.0013 (12)	0.0029 (13)	0.0114 (13)
C9	0.0449 (16)	0.0508 (16)	0.0395 (16)	0.0082 (13)	0.0032 (12)	0.0066 (13)
C10	0.060 (2)	0.083 (2)	0.0459 (19)	0.0155 (18)	0.0124 (15)	0.0165 (17)
C11	0.082 (3)	0.103 (3)	0.0357 (18)	0.020 (2)	0.0081 (17)	−0.011 (2)
C12	0.084 (2)	0.075 (2)	0.047 (2)	0.0125 (19)	0.0021 (17)	−0.0225 (18)
C13	0.071 (2)	0.0479 (17)	0.0427 (17)	0.0051 (15)	0.0013 (14)	−0.0102 (14)
C14	0.0477 (15)	0.0429 (15)	0.0305 (14)	0.0059 (12)	0.0016 (12)	−0.0033 (12)
C15	0.066 (2)	0.0318 (14)	0.0467 (17)	−0.0015 (13)	−0.0036 (14)	−0.0077 (12)
N2	0.0670 (17)	0.0384 (13)	0.0434 (14)	0.0101 (12)	0.0046 (12)	−0.0085 (11)
C16	0.0510 (17)	0.0538 (17)	0.0376 (16)	0.0071 (14)	0.0004 (13)	−0.0008 (13)
C17	0.064 (2)	0.087 (3)	0.050 (2)	0.0044 (19)	0.0009 (16)	0.0105 (19)
C18	0.070 (3)	0.165 (5)	0.036 (2)	0.011 (3)	0.0040 (17)	−0.011 (3)
C19	0.069 (3)	0.130 (4)	0.076 (3)	0.009 (3)	−0.004 (2)	−0.053 (3)
C20	0.082 (3)	0.079 (3)	0.077 (3)	−0.003 (2)	−0.002 (2)	−0.030 (2)
C21	0.073 (2)	0.057 (2)	0.054 (2)	0.0000 (16)	0.0075 (17)	−0.0085 (16)
N1	0.0539 (14)	0.0284 (11)	0.0333 (12)	0.0019 (9)	0.0017 (10)	−0.0019 (9)
O1	0.0563 (12)	0.0433 (10)	0.0378 (11)	0.0081 (9)	0.0116 (9)	0.0007 (8)
O2	0.0572 (12)	0.0313 (9)	0.0611 (13)	−0.0067 (8)	0.0115 (10)	−0.0007 (9)
O3	0.0705 (15)	0.0824 (17)	0.0664 (15)	0.0217 (13)	0.0144 (12)	−0.0178 (13)
O4	0.103 (2)	0.0382 (12)	0.0928 (19)	0.0131 (12)	−0.0088 (15)	0.0084 (12)
S1	0.0483 (4)	0.0282 (3)	0.0369 (4)	0.0012 (3)	0.0085 (3)	0.0019 (3)
S2	0.0689 (5)	0.0416 (4)	0.0516 (5)	0.0154 (4)	0.0018 (4)	−0.0060 (3)
Br1	0.0843 (3)	0.0436 (2)	0.0925 (3)	−0.00243 (16)	0.0188 (2)	0.02590 (17)

### Geometric parameters (Å, º)

**Table:**

C1—C6	1.371 (4)	C13—C14	1.386 (4)
C1—C2	1.393 (4)	C13—H13	0.9300
C1—S1	1.748 (3)	C14—N1	1.418 (3)
C2—C3	1.372 (4)	C15—N2	1.453 (4)
C2—H2	0.9300	C15—H15A	0.9700
C3—C4	1.377 (5)	C15—H15B	0.9700
C3—H3	0.9300	N2—S2	1.627 (3)
C4—C5	1.369 (5)	N2—H2A	0.874 (10)
C4—H4	0.9300	C16—C17	1.364 (5)
C5—C6	1.378 (4)	C16—C21	1.373 (4)
C5—H5	0.9300	C16—S2	1.764 (3)
C6—H6	0.9300	C17—C18	1.391 (6)
C7—C8	1.336 (4)	C17—H17	0.9300
C7—N1	1.421 (3)	C18—C19	1.361 (7)
C7—C15	1.502 (4)	C18—H18	0.9300
C8—C9	1.426 (4)	C19—C20	1.346 (7)
C8—Br1	1.868 (3)	C19—H19	0.9300
C9—C14	1.387 (4)	C20—C21	1.363 (5)
C9—C10	1.388 (4)	C20—H20	0.9300
C10—C11	1.367 (5)	C21—H21	0.9300
C10—H10	0.9300	N1—S1	1.659 (2)
C11—C12	1.380 (5)	O1—S1	1.4203 (19)
C11—H11	0.9300	O2—S1	1.416 (2)
C12—C13	1.370 (4)	O3—S2	1.419 (3)
C12—H12	0.9300	O4—S2	1.421 (3)
			
C6—C1—C2	121.7 (3)	N2—C15—C7	116.1 (2)
C6—C1—S1	119.4 (2)	N2—C15—H15A	108.3
C2—C1—S1	118.8 (2)	C7—C15—H15A	108.3
C3—C2—C1	118.4 (3)	N2—C15—H15B	108.3
C3—C2—H2	120.8	C7—C15—H15B	108.3
C1—C2—H2	120.8	H15A—C15—H15B	107.4
C2—C3—C4	120.2 (3)	C15—N2—S2	122.6 (2)
C2—C3—H3	119.9	C15—N2—H2A	113 (2)
C4—C3—H3	119.9	S2—N2—H2A	110 (2)
C5—C4—C3	120.7 (3)	C17—C16—C21	121.1 (3)
C5—C4—H4	119.6	C17—C16—S2	120.6 (3)
C3—C4—H4	119.6	C21—C16—S2	118.3 (2)
C4—C5—C6	120.2 (3)	C16—C17—C18	118.1 (4)
C4—C5—H5	119.9	C16—C17—H17	121.0
C6—C5—H5	119.9	C18—C17—H17	121.0
C1—C6—C5	118.8 (3)	C19—C18—C17	120.0 (4)
C1—C6—H6	120.6	C19—C18—H18	120.0
C5—C6—H6	120.6	C17—C18—H18	120.0
C8—C7—N1	107.2 (2)	C20—C19—C18	121.2 (4)
C8—C7—C15	128.6 (2)	C20—C19—H19	119.4
N1—C7—C15	123.6 (2)	C18—C19—H19	119.4
C7—C8—C9	110.9 (2)	C19—C20—C21	119.9 (4)
C7—C8—Br1	125.5 (2)	C19—C20—H20	120.1
C9—C8—Br1	123.5 (2)	C21—C20—H20	120.1
C14—C9—C10	119.8 (3)	C20—C21—C16	119.7 (4)
C14—C9—C8	106.4 (2)	C20—C21—H21	120.1
C10—C9—C8	133.7 (3)	C16—C21—H21	120.1
C11—C10—C9	118.4 (3)	C14—N1—C7	107.8 (2)
C11—C10—H10	120.8	C14—N1—S1	122.48 (17)
C9—C10—H10	120.8	C7—N1—S1	126.06 (17)
C10—C11—C12	121.3 (3)	O2—S1—O1	119.71 (12)
C10—C11—H11	119.4	O2—S1—N1	105.73 (12)
C12—C11—H11	119.4	O1—S1—N1	106.35 (11)
C13—C12—C11	121.5 (3)	O2—S1—C1	108.96 (12)
C13—C12—H12	119.3	O1—S1—C1	108.32 (12)
C11—C12—H12	119.3	N1—S1—C1	107.10 (12)
C12—C13—C14	117.3 (3)	O3—S2—O4	120.92 (16)
C12—C13—H13	121.3	O3—S2—N2	105.18 (15)
C14—C13—H13	121.3	O4—S2—N2	106.73 (15)
C13—C14—C9	121.7 (3)	O3—S2—C16	107.51 (15)
C13—C14—N1	130.6 (3)	O4—S2—C16	108.14 (16)
C9—C14—N1	107.7 (2)	N2—S2—C16	107.71 (13)
			
C6—C1—C2—C3	0.2 (4)	C18—C19—C20—C21	0.8 (7)
S1—C1—C2—C3	177.5 (2)	C19—C20—C21—C16	−0.8 (6)
C1—C2—C3—C4	−1.0 (5)	C17—C16—C21—C20	0.5 (5)
C2—C3—C4—C5	1.2 (5)	S2—C16—C21—C20	178.2 (3)
C3—C4—C5—C6	−0.5 (5)	C13—C14—N1—C7	178.7 (3)
C2—C1—C6—C5	0.4 (4)	C9—C14—N1—C7	0.4 (3)
S1—C1—C6—C5	−176.9 (2)	C13—C14—N1—S1	−21.7 (4)
C4—C5—C6—C1	−0.3 (5)	C9—C14—N1—S1	159.99 (19)
N1—C7—C8—C9	−0.2 (3)	C8—C7—N1—C14	−0.1 (3)
C15—C7—C8—C9	171.5 (3)	C15—C7—N1—C14	−172.3 (2)
N1—C7—C8—Br1	−175.46 (19)	C8—C7—N1—S1	−158.8 (2)
C15—C7—C8—Br1	−3.8 (4)	C15—C7—N1—S1	29.0 (4)
C7—C8—C9—C14	0.5 (3)	C14—N1—S1—O2	45.3 (2)
Br1—C8—C9—C14	175.8 (2)	C7—N1—S1—O2	−158.9 (2)
C7—C8—C9—C10	−175.7 (3)	C14—N1—S1—O1	173.6 (2)
Br1—C8—C9—C10	−0.3 (5)	C7—N1—S1—O1	−30.7 (3)
C14—C9—C10—C11	2.1 (5)	C14—N1—S1—C1	−70.8 (2)
C8—C9—C10—C11	177.8 (3)	C7—N1—S1—C1	85.0 (2)
C9—C10—C11—C12	−0.4 (5)	C6—C1—S1—O2	151.8 (2)
C10—C11—C12—C13	−1.2 (6)	C2—C1—S1—O2	−25.6 (2)
C11—C12—C13—C14	1.2 (5)	C6—C1—S1—O1	20.0 (3)
C12—C13—C14—C9	0.6 (4)	C2—C1—S1—O1	−157.4 (2)
C12—C13—C14—N1	−177.5 (3)	C6—C1—S1—N1	−94.3 (2)
C10—C9—C14—C13	−2.2 (4)	C2—C1—S1—N1	88.3 (2)
C8—C9—C14—C13	−179.0 (3)	C15—N2—S2—O3	174.6 (2)
C10—C9—C14—N1	176.3 (3)	C15—N2—S2—O4	45.0 (3)
C8—C9—C14—N1	−0.5 (3)	C15—N2—S2—C16	−70.9 (2)
C8—C7—C15—N2	−114.1 (3)	C17—C16—S2—O3	−136.9 (3)
N1—C7—C15—N2	56.3 (4)	C21—C16—S2—O3	45.3 (3)
C7—C15—N2—S2	66.0 (3)	C17—C16—S2—O4	−4.8 (3)
C21—C16—C17—C18	−0.2 (5)	C21—C16—S2—O4	177.4 (3)
S2—C16—C17—C18	−177.9 (3)	C17—C16—S2—N2	110.2 (3)
C16—C17—C18—C19	0.2 (6)	C21—C16—S2—N2	−67.5 (3)
C17—C18—C19—C20	−0.5 (7)		

### Hydrogen-bond geometry (Å, º)

Cg2 is the centroid of the C1–C6 ring.

**Table:**

*D*—H···*A*	*D*—H	H···*A*	*D*···*A*	*D*—H···*A*
N2—H2*A*···O1	0.87 (1)	2.17 (3)	2.795 (3)	128 (3)
C13—H13···O2	0.93	2.38	2.954 (4)	120
C21—H21···*Cg*2^i^	0.93	2.81	3.667 (3)	155

Symmetry code: (i) *x*+1, *y*, *z*.

## References

[bb1] Andreani, A., Granaiola, M., Leoni, A., Locatelli, A., Morigi, R., Rambaldi, M., Giorgi, G., Salvini, L. & Garaliene, V. (2001). *Anticancer Drug. Des.* **16**, 167–174. 11962514

[bb2] Andreev, I. A., Manvar, D., Barreca, M. L., Belov, D. S., Basu, A., Sweeney, N. L., Ratmanova, N. K., Lukyanenko, E. R., Manfroni, G., Cecchetti, V., Frick, D. N., Altieri, A., Kaushik-Basu, N. & Kurkin, A. V. (2015). *Eur. J. Med. Chem.* **96**, 250–258. 10.1016/j.ejmech.2015.04.022 PMC5557381 25890075

[bb3] Bruker (2004). *APEX2* and *SAINT*. Bruker AXS Inc., Madison, Wisconsin, USA.

[bb4] Chakkaravarthi, G., Dhayalan, V., Mohanakrishnan, A. K. & Manivannan, V. (2008). *Acta Cryst.* E**64**, o542. 10.1107/S1600536808003024 PMC2960400 21201561

[bb5] Chakkaravarthi, G., Ramesh, N., Mohanakrishnan, A. K. & Manivannan, V. (2007). *Acta Cryst.* E**63**, o3564.

[bb6] Kolocouris, N., Foscolos, G. B., Kolocouris, A., Marakos, P., Pouli, N., Fytas, G., Ikeda, S. & De Clercq, E. (1994). *J. Med. Chem.* **37**, 2896–2902. 10.1021/jm00044a010 8071937

[bb7] Sheldrick, G. M. (1996). *SADABS*. University of Göttingen, Germany.

[bb8] Sheldrick, G. M. (2008). *Acta Cryst.* A**64**, 112–122. 10.1107/S0108767307043930 18156677

[bb9] Spek, A. L. (2009). *Acta Cryst.* D**65**, 148–155. 10.1107/S090744490804362X PMC2631630 19171970

