# Supplementary figures and images for: Crystal structure of N-{[3-bromo-1-(phenyl­sulfon­yl)-1H-indol-2-yl]meth­yl}benzene­sulfonamide
Source: Acta Crystallogr E Crystallogr Commun. 2015 Sep 17;71(Pt 10):o756–7. doi: 10.1107/S2056989015016874 (PMC4647446; doi:10.1107/S2056989015016874)

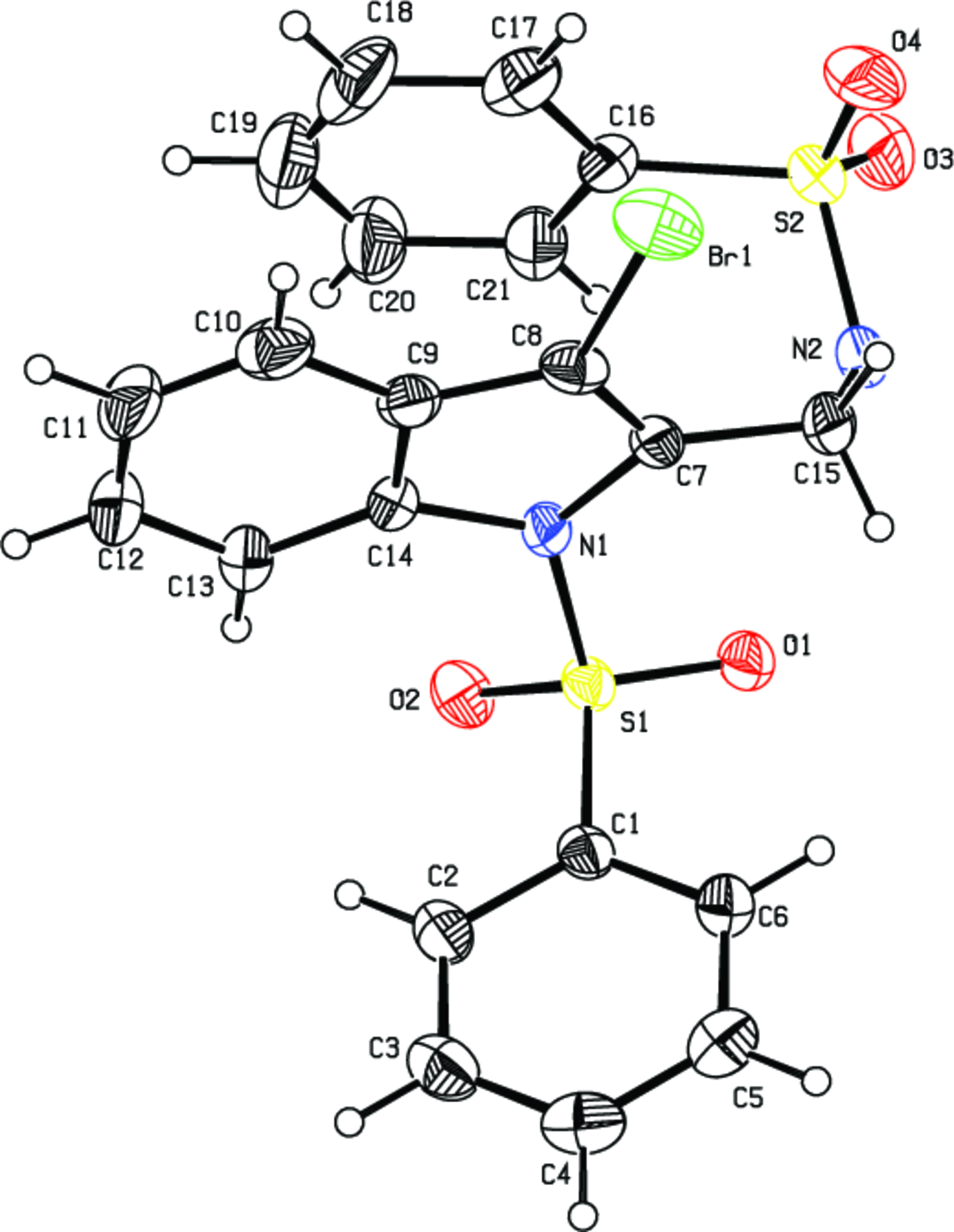

Supplement: Supplementary file 4 [file e-71-0o756-fig1.tif]
